# Short‐duration intermittent hypoxia enhances endurance capacity by improving muscle fatty acid metabolism in mice

**DOI:** 10.14814/phy2.12744

**Published:** 2016-04-06

**Authors:** Junichi Suzuki

**Affiliations:** ^1^ Laboratory of Exercise PhysiologyHealth and Sports SciencesCourse of Sports EducationDepartment of EducationHokkaido University of Education, MidorigaokaIwamizawaHokkaido068‐8642Japan

**Keywords:** Capillarization, exercise training, fatty acid metabolism, intermittent hypoxia, microRNA

## Abstract

This study was designed to (1) investigate the effects of acute short‐duration intermittent hypoxia on muscle mRNA and microRNA expression levels; and (2) clarify the mechanisms by which short‐duration intermittent hypoxia improves endurance capacity. Experiment‐1: Male mice were subjected to either acute 1‐h hypoxia (12% O_2_), acute short‐duration intermittent hypoxia (12% O_2_ for 15 min, room air for 10 min, 4 times, Int‐Hypo), or acute endurance exercise (Ex). The expression of vascular endothelial growth factor‐A mRNA was significantly greater than the control at 0 h post Ex and 6 h post Int‐Hypo in the deep red region of the gastrocnemius muscle. miR‐16 expression levels were significantly lower at 6 and 10 h post Int‐Hypo. Peroxisome proliferator‐activated receptor gamma coactivator 1‐alpha (PGC‐1*α*) mRNA levels were significantly greater than the control at 3 h post Ex and 6 h post Int‐Hypo. miR‐23a expression levels were lower than the control at 6–24 h post Int‐Hypo. Experiment‐2: Mice were subjected to normoxic exercise training with or without intermittent hypoxia for 3 weeks. Increases in maximal exercise capacity were significantly greater by training with short‐duration intermittent hypoxia (IntTr) than without hypoxia. Both 3‐Hydroxyacyl‐CoA‐dehydrogenase and total carnitine palmitoyl transferase activities were significantly enhanced in IntTr. Peroxisome proliferator‐activated receptor delta and PGC‐1*α* mRNA levels were both significantly greater in IntTr than in the sedentary controls. These results suggest that exercise training under normoxic conditions with exposure to short‐duration intermittent hypoxia represents a beneficial strategy for increasing endurance performance by enhancing fatty acid metabolism in skeletal muscle.

## Introduction

Exercise training at high altitude was developed in the early 1990s in order to enhance exercise performance. Using artificial hypoxic apparatuses or high altitude environments, several forms of training have hitherto been established in an attempt to improve athletic performance at sea level or high altitude, for example, “live low‐train high” and “live high‐train low.” Although numerous combinations of the duration of hypoxic exposure, intensity of exercise, and period of training have been experimentally investigated, an essential strategy to improve athletic performance, especially endurance capacity at sea level, has not yet been clearly identified (Terrados et al. 1990; Levine et al. 1992; Vogt et al. 2001; Julian et al. 2004; Katayama et al. 2004; Bakkman et al. 2007; Vogt and Hoppeler 2010; De Paula and Niebauer 2012).

Intermittent hypoxic training comprises several hours or overnight hypoxic exposure at rest per day with exercise training under normoxia, and has been proposed as a useful strategy that does not restrict the daily training regimens of athletes (Julian et al. 2004; Katayama et al. 2004). Furthermore, repeated short‐duration hypoxic exposure, that is, several minutes of hypoxia interspersed with several minutes of normoxia, has been developed in order to allow a large number of athletes to experience a hypoxic atmosphere. However, the effects of hypoxic exposure, comprising a 3–6‐min hypoxia/normoxia cycle, on exercise performance remain controversial in humans (Julian et al. 2004; Hinckson et al. 2007; Bärtsch et al. 2008; Bonetti et al. 2009; Mekjavic et al. 2012).

Drevytska et al. (2012) reported that normoxic exercise training by swimming in combination with short‐duration intermittent hypoxia at rest (12% O_2_ for 15 min, room air for 15 min, 5 times per day) for 2 weeks markedly improved swimming time to exhaustion and maximal oxygen consumption in rats. However, the underlying mechanisms for the improvements induced in endurance capacity by short‐duration intermittent hypoxia have not yet been elucidated in detail.

In this study, experiments were designed to (1) investigate the effects of acute short‐duration intermittent hypoxia on muscle mRNA and microRNA expression levels; and (2) clarify how short‐duration intermittent hypoxia has additive effects on improvements to endurance capacity during exercise training.

## Materials and methods

### Ethical approval

All procedures were approved by the Animal Care and Use Committee of Hokkaido University of Education and performed in accordance with the “Guiding Principles for the Care and Use of Animals in the Field of Physiological Sciences” of the Physiological Society of Japan.

### Animals

Male ICR mice (10 weeks old) were purchased from Clea Japan Inc. (Tokyo, Japan) and housed under conditions of a controlled temperature (24 ± 1°C) and relative humidity of approximately 50%. Lighting (7:00–19:00) was controlled automatically. All mice were given commercial laboratory chow (solid CE‐2, Clea Japan) and tap water ad libitum. After mice had been fed for 2 weeks and allowed to adapt to the new environment, they were assigned to each experiment. During the second week of the adaptation period, all mice were subjected to treadmill walking using a rodent treadmill (KN‐73, Natsume Co., Tokyo, Japan) for 10 min per day at 10 m min^−1^ with a 0% grade for 3 days.

### Experiment 1: Time‐course response to acute hypoxic exposure or exercise

Sixty‐five male mice were randomly assigned to a sedentary control group (Cnt, *n* = 5), acutely hypoxic exposed group (1H‐Hypo, *n* = 20), acutely intermittent hypoxic exposed group (Int‐Hypo, *n* = 20), or acutely exercised group (Ex, *n* = 20). Normobaric hypoxia was achieved in the 1H‐Hypo group by breathing a hypoxic gaseous mixture containing 12% O_2_ for 1 h. The Int‐Hypo group was subjected to intermittent hypoxic exposure, which consisted of four cycles of hypoxic air (12% O_2_) breathing for 15 min and room air (20.9% O_2_) breathing for 10 min. The Ex group was subjected to acute endurance exercise for 1 h at 20 m min^−1^ with a 10% incline. Following each treatment, tissues were collected at either 0 (immediately post treatment, *n* = 4), 3 (*n* = 4), 6 (*n* = 4), 10 (*n* = 4), or 24 (*n* = 4) h. Mice were anesthetized with *α*‐chloralose (0.06 g kg^−1^ i. p.) and urethane (0.7 g kg^−1^ i. p.). A toe pinch response was used to validate adequate anesthesia. The gastrocnemius muscle and kidney was excised and weighed. All tissue samples were frozen in liquid nitrogen and stored at −80°C until later analyses.

### RNA isolation and cDNA synthesis

The mRNA‐containing and microRNA fractions were isolated using RNAzol RT (Molecular Research Center, Inc., Ohio). In order to determine mRNA, 4.5 *μ*g of the mRNA‐containing fraction was taken for cDNA synthesis using an oligo dT primer and Mmlv reverse transcriptase (RNase H minus point mutant, ReverTra Ace, Toyobo Co., Tokyo, Japan). Poly(A)‐tails were added to the 3′ end of the microRNA fraction using the A‐Plus Poly(A) Polymerase Tailing Kit (Cellscript Inc., Wisconsin) in order to determine microRNA. cDNA was then synthesized using an oligo dT primer with the adaptor sequence 5′‐GGCCACGCGTCGACTAGTACTTTTTTTTTTTTTTTTT‐3′ and reverse transcriptase (ReverTra Ace).

### Real‐time PCR analyses

mRNA and microRNA levels were determined by a standard real‐time polymerase chain reaction using the KAPA SYBR FAST qPCR Kit (KAPA Biosystems Inc., Massachusetts). Hypoxanthine ribosyltransferase (HPRT) and retention in endoplasmic reticulum‐1 (Rer‐1), and small nuclear RNA U6 (snoU6) were used as endogenous controls for mRNA and microRNA expression analyses, respectively. The PCR conditions for mRNA were: 1 min predenaturation at 95°C, and then 10 sec denaturation at 95°C, 20 sec annealing at 60, 61, or 62°C, and 1 sec extension at 70°C for 40 cycles. The following protocol was used to amplify microRNA: 1 min predenaturation at 95°C, and then 3 sec denaturation at 95°C and 20 sec annealing/extension at 60 or 62°C for 40 cycles. A real‐time analysis of PCR amplification was performed on a CFX96 real‐time PCR system and analyzed with the CFX Manager software (Bio‐Rad Inc., California). Serial fivefold dilutions of a cDNA sample were used to generate a standard curve. Nonspecific products such as primer dimer formation were checked by dissociation curves and the results of negative control samples without cDNA. The sequences of the forward and reverse primer sets used for PCR amplification of the various genes and microRNAs are shown in Table 1.

**Table 1:** Sequences of forward and reverse primer sets used for PCR amplification of genes and microRNAs

Target	Forward primer (5′ to 3′)	Forward primer (5′ to 3′)	GenBank accession no.
VEGF‐A	GCACTGGACCCTGGCTTTACTGCTG	ACGGCAATAGCTGCGCTGGTAGAC	NM_001025250.3
PGC‐1*α*	TCCTCACACCAAACCCACAGA	AACCCTTGGGGTCATTTGGTGA	NM_008904.2
PPAR*δ*	GGGAAAAGTTTTGGCAGGAGC	CAGATGGACTGCCTTTACCGTG	NM_011145.3
EPO	ATGTCACGATGGGTTGTGCAG	TGGCTGGGAGGAATTGGCTA	NM_007942.2
RER‐1	ACCGGAGCTGCGAGTTACAGAA	TAGACTTGTCCAGCCAGGACTGA	NM_026395.1
HPRT	CGACCCTCAGTCCCAGCGTCGTGATTA	AGGGCCACAATGTGATGGCCTCCCA	NM_013556.2
Target	Mature sequence (5′ to 3′)	Forward primer (5′ to 3′)	miRBase or GenBank accession no.
mmu‐miR‐16	UAGCAGCACGUAAAUAUUGGCG	TACGATAGCAGCACGTAAATATTGGCG	MIMAT0000527
mmu‐miR‐23a	AUCACAUUGCCAGGGAUUUCC	CGCATCACATTGCCAGGGATTTCC	MIMAT0000532
mmu‐snoU6	GTGCTCGCTTCGGCAGCACATATACTAA AATTGGAACGATACAGAGAAGATTAGCA TGGCCCCTGCGCAAGGATGACACGCAA ATTCGTGAAGCGTTCCATATTTTT	TGGCCCCTGCGCAAGGATG	NR_003027.1
Universal reverse primer: GGCCACGCGTCGACTAGTAC			

VEGF‐A, vascular endothelial growth factor‐A; PGC‐1*α*, peroxisome proliferator‐activated receptor gamma coactivator 1‐alpha; PPAR*δ*, peroxisome proliferator‐activated receptor delta; EPO, erythropoietin; RER‐1, retention in endoplasmic reticulum‐1; HPRT, hypoxanthine ribosyltransferase; snoU6, small nuclear RNA U6.

### Experiment 2: Chronic response of exercise training with hypoxic exposure

**Figure 1:**
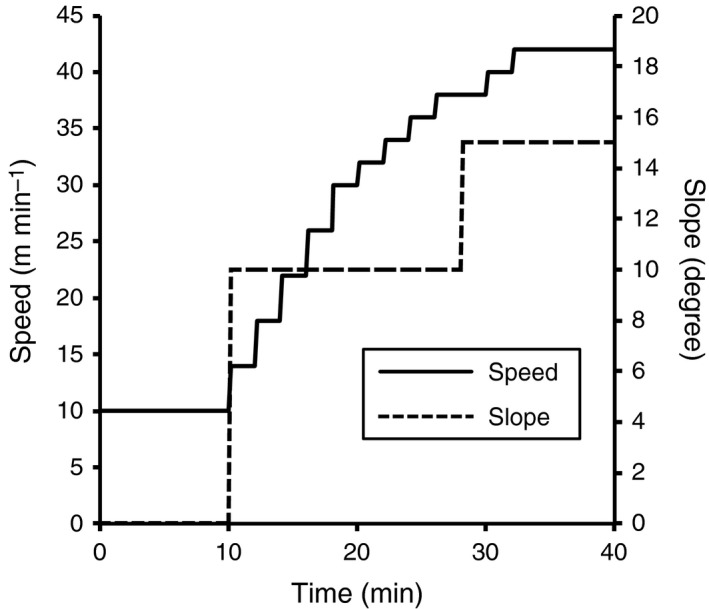
Graded ramp treadmill running protocol.

Seventy‐two male mice were randomly assigned into the following six groups: sedentary controls (Cnt, *n* = 12), the 1‐h hypoxic exposure group (1H, *n* = 12), intermittent hypoxic exposure group (Int, *n* = 12), normoxic exercise‐trained group (Tr, *n* = 12), 1‐h hypoxic exposure with normoxic exercise‐trained group (1HTr, *n* = 12), and intermittent hypoxic exposure with normoxic exercise‐trained group (IntTr, *n* = 12). Intermittent or 1‐h hypoxic exposure was performed as described for Experiment 1, once per day, 5 days per week, for 3 weeks. The 1HTr and IntTr groups were exposed to 1 h hypoxia or intermittent hypoxia, respectively, followed by daily exercise training under normoxic conditions, as described below. Mice in the training groups were subjected to endurance exercise training for 3 weeks, 5 days per week. Mice ran for 60 min at 20 m min^−1^ with a 15% grade on the first day of training. The speed was gradually increased to 25 m min^−1^ throughout the training period. Twenty‐four hours after the last training bout, maximal exercise capacity was determined using a graded ramp treadmill running protocol, as shown in Figure 1. The test was performed using a controlled treadmill (Modular motor assay, Columbus Instruments Inc., Columbus, OH).

Mice were anesthetized as described for Experiment 1. The right gastrocnemius muscle was excised and weighed. The deep red region (Gr) was isolated from the superficial white region (Gw) and then frozen in liquid nitrogen for biochemical analyses. The remaining muscle, that is,. the right side, was excised and placed in embedding medium, O.C.T. compound (Miles Inc., Elkhart, IN), and then rapidly frozen in isopentane cooled to its melting point (−160°C) with liquid nitrogen. Blood samples were collected from the beating heart, and hemoglobin concentrations were measured spectrophotometrically (Hemoglobin‐kit, Wako Pure Chemical Industries, Ltd., Osaka, Japan). The heart was excised, weighed, and frozen in liquid nitrogen. All tissue samples were stored at −80°C until later analyses.

### Histological analyses

Serial 10‐micrometer‐thick cross sections were obtained using a cryotome (CM‐1500; Leica Japan Inc., Tokyo, Japan) at −20°C from the mid‐belly portion of the gastrocnemius muscle. These sections were air‐dried, fixed with acetone at 4°C, and then washed in 0.1 mol L^−1^ phosphate‐buffered saline with 0.05% Triton X‐100 (PBS‐T). In order to determine capillary profiles, the sections were incubated overnight at 4°C with fluorescein‐labeled Griffonia simplicifolia lectin (GSL I) (FL 1101 (1:300), Vector Laboratories Inc., California)(Hansen‐Smith et al. 1992). To determine muscle fiber phenotypes, sections were blocked with 10% goat normal serum and then incubated overnight at 4°C with antifast myosin (M4276, Sigma‐Aldrich Co., Missouri) or antislow myosin (M8421, Sigma‐Aldrich) antibodies diluted 1:1000 with PBS‐T containing 5% goat normal serum. After washing three times with PBS‐T, the sections were reacted with an Alexa Fluor 647‐labeled secondary antibody (1:1000, ab150119, Abcam Japan, Tokyo, Japan) for 1 h at room temperature. The sections were washed three times with PBST, air‐dried, and coverslipped with Fluoromount/Plus (K048, Diagnostic BioSystems Co., California). Fluorescent images of the incubated sections were observed using a microscope (Axio Observer, Carl Zeiss Japan, Tokyo, Japan) and stored on a computer disk. Muscle capillary, fiber phenotype, and cross‐sectional area were assessed in Gr and Gw. The fibers were classified as slow, fast, or the slow + fast hybrid. Nonoverlapping microscopic fields were selected at random from each muscle sample. The observer was blind to the source (groups) of each slide during the measurements.

### Biochemical analyses of enzyme activity

Frozen tissue powder was obtained using a frozen sample crusher (SK mill, Tokken Inc., Chiba, Japan) and homogenized with ice‐cold medium (10 mmol L^−1^ HEPES buffer, pH 7.3; 0.1% Triton X‐100; 11.5% (w v^−1^) sucrose; and 5% (v v^−1^) protease inhibitor cocktail [P2714, Sigma‐Aldrich Co., Missouri]). After centrifugation at 1500 × *g* at 4°C for 10 min, the supernatant was used in enzyme activity analyses. The activities of 3‐hydroxyacyl‐CoA‐dehydrogenase (HAD) and lactate dehydrogenase (LDH) were assayed according to the method of Bass et al. (1969). The activities of citrate synthase (CS) and phosphofructokinase (PFK) were determined according to the method of Srere (1969) and Passonneau and Lowry (1993), respectively. Total carnitine palmitoyl transferase (CPT) activity was assayed according to the method of Abel (2004). All measurements were conducted at 25°C with a spectrophotometer (U‐2001, Hitachi Co., Tokyo, Japan) and enzyme activities were obtained as *μ*mol h^−1^ g protein^−1^. Total protein concentrations were measured using PRO‐MEASURE protein measurement solution (iNtRON Biotechnology Inc., Gyeonggi‐do, Korea).

### Statistical analyses

All values are transformed using base‐10 logs. Differences among the groups were analyzed using a one‐way analysis of variance (ANOVA). When a significant difference was observed, Dunnett's and Tukey's multiple comparison procedures were used to determine group differences in experiments 1 and 2, respectively. Differences were considered significant at *P* < 0.05 and 95% confidential interval (CI) did not contain the parameter value specified in the null hypothesis. Because the sample size was small in the experiment 1, the 95% CI values were expressed in the results to show the power and precision of the experiment as well as the likely physiological importance of the results. Mean, standard deviation (SD), and CI values are expressed after back‐transformation. All statistics were performed using Statview software (v5.0, SAS Institute Inc., Cary, NC).

## Results

### Experiment 1: Time‐course response to acute hypoxic exposure or exercise

**Figure 2:**
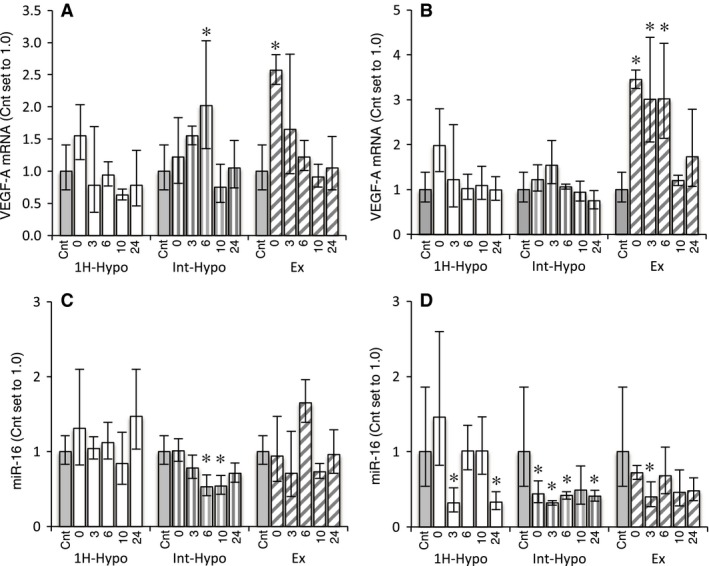
Expression of VEGF‐A mRNA (A, B) and miR‐16 (C, D) in the red (A, C) and white (B, D) regions of the gastrocnemius muscle. Values are represented as means ± SD. The number of mice was 4 per group, except for Cnt (*n* = 5). *, significantly different from the Cnt group at *P* *<* 0.05 using a one‐way ANOVA followed by Dunnett's post hoc test and 95% confidential interval did not contain 1.0.

The expression of VEGF‐A mRNA in Gr was significantly greater than the control at 0 h post Ex (95% CI: 2.2–3.0, *P* < 0.05) and 6 h post Int‐Hypo (95% CI: 1.1–3.9, *P* < 0.05, Fig. 2A). VEGF‐A mRNA levels in Gw were greater than the control at 0 (95% CI: 3.1–3.8, *P* < 0.05), 3 (95% CI: 1.6–5.5, *P* < 0.05), and 6 h (95% CI: 1.7–5.2, *P* < 0.05) post Ex (Fig. 2B). The expression levels of miR‐16 in Gr were markedly lower at 6 (95% CI: 0.35–0.81, *P* < 0.05) and 10 h (95% CI: 0.38–0.78, *P* < 0.05) post Int‐Hypo (Fig. 2C). miR‐16 levels in Gw were significantly lower than the control at 3 (95% CI: 0.14–0.70, *P* < 0.05) and 24 h (95% CI: 0.19–0.58, *P* < 0.05) post 1H, 0 (95% CI: 0.26–0.74, *P* < 0.05), 3 (95% CI: 0.28–0.37, *P* < 0.05), and 24 h (95% CI: 0.31–0.55, *P* < 0.05) post Int‐Hypo, and 3 h post Ex (95% CI: 0.21–0.77, *P* < 0.05, Fig. 2D).

**Figure 3:**
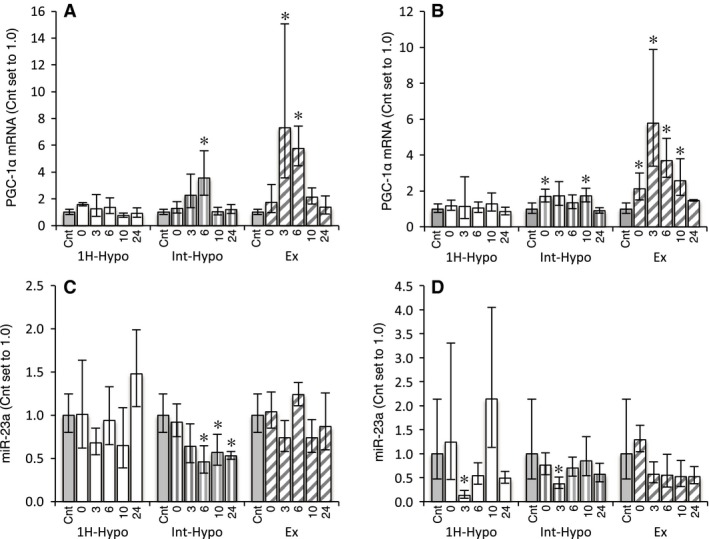
Expression of PGC‐1*α* mRNA (A, B) and miR‐23a (C, D) in the red (A, C) and white (B, D) regions of the gastrocnemius muscle. Values are represented as means ± SD. The number of mice was 4 per group, except for Cnt (*n* = 5). *, significantly different from the Cnt group at *P* *<* 0.05 using a one‐way ANOVA followed by Dunnett's post hoc test and 95% confidential interval did not contain 1.0.

PGC‐1*α* mRNA levels in Gr were significantly greater than the control at 3 (95% CI: 2.3–23.0, *P* < 0.05) and 6 h (95% CI: 3.8–8.6, *P* < 0.05) post Ex and 6 h post Int‐Hypo (95% CI: 1.7–7.3, *P* < 0.05, Fig. 3A). PGC‐1*α* expression levels in Gw were greater at 0 (95% CI: 1.2–3.7, *P* < 0.05), 3 (95% CI: 2.5–13.6, *P* < 0.05), 6 (95% CI: 2.3–5.9, *P* < 0.05) and 10 h (95% CI: 1.4–4.8, *P* < 0.05) post Ex and 0 (95% CI: 1.2–2.4, *P* < 0.05) and 10 h (95% CI: 1.2–2.4, *P* < 0.05) post Int‐Hypo (Fig. 3B). The expression levels of miR‐23a were significantly lower than the control at 6 (95% CI: 0.27–0.80, *P* < 0.05), 10 (95% CI: 0.35–0.94, *P* < 0.05), and 24 h (95% CI: 0.46–0.61, *P* < 0.05) post Int‐Hypo in Gr and at 3 h post 1H (95% CI: 0.05–0.33, *P* < 0.05) and 3 h post Int‐Hypo (95% CI: 0.23–0.62, *P* < 0.05) in Gw (Fig. 3C and D).

In the kidney, EPO mRNA levels were significantly greater than the control at 0 h post all three treatments (95% CI: 6.6–35.9 for 1H, 2.5–22.2 for Int‐Hypo, and 3.0–12.6 for Ex, *P* < 0.05).

### Experiment 2: Chronic response of exercise training with hypoxic exposure

#### Body and organ masses and maximal exercise capacity

The body and organ weights of each group were not significantly different among the groups (Table 2). No significant differences were observed in hemoglobin concentrations after training and/or hypoxic exposure.

**Table 2:** Body and organ masses

	Cnt	Tr	1H	1HTr	Int	IntTr
Body mass (g)	41.2 ± 2.1/2.0	40.2 ± 1.4/1.4	41.6 ± 3.5/3.2	40.8 ± 2.5/2.4	42.0 ± 1.8/1.7	40.6 ± 2.1/2.0
Organ mass (mg)						
Gastrocnemius	172.9 ± 26.0/22.6	181.8 ± 13.2/12.3	178.0 ± 18.4/16.7	184.6 ± 15.1/13.9	192.9 ± 13.9/12.9	178.1 ± 10.5/10.0
Whole heart	172.9 ± 18.0/16.3	181.6 ± 14.5/13.4	172.4 ± 7.6/7.3	180.2 ± 13.8/12.8	169.8 ± 13.1/12.1	183.3 ± 9.8/9.3
Left ventricle	121.8 ± 14.0/12.6	125.8 ± 10.8/10.0	120.6 ± 5.8/5.5	127.8 ± 12.7/11.5	119.0 ± 8.7/8.1	129.0 ± 8.5/8.0
Kidney	687.5 ± 73.2/66.1	713.2 ± 73.6/66.7	643.5 ± 95.3/83.0	677.7 ± 82.1/73.2	625.1 ± 65.3/59.1	691.6 ± 55.1/51.0
Organ mass‐to‐body mass ratio (mg g^−1^)						
Gastrocnemius	4.20 ± 0.54/0.48	4.50 ± 0.24/0.22	4.28 ± 0.53/0.47	4.53 ± 0.48/0.43	4.62 ± 0.36/0.33	4.38 ± 0.35/0.33
Whole heart	4.20 ± 0.26/0.25	4.49 ± 0.33/0.31	4.14 ± 0.38/0.35	4.42 ± 0.16/0.15	4.05 ± 0.36/0.33	4.51 ± 0.21/0.20
Left ventricle	2.96 ± 0.21/0.20	3.13 ± 0.24/0.23	2.90 ± 0.28/0.25	3.12 ± 0.19/0.18	2.83 ± 0.25/0.23	3.19 ± 0.20/0.18
Kidney	16.7 ± 1.3/1.2	17.7 ± 2.2/2.0	15.5 ± 2.1/1.9	16.6 ± 1.3/1.2	14.9 ± 1.6/1.5	17.0 ± 1.1/1.0
Hemoglobin (g 100 mL^−1^)	14.5 ± 1.6/1.4	13.8 ± 1.6/1.5	13.6 ± 1.8/1.6	14.1 ± 2.0/1.7	14.6 ± 0.95/0.89	13.9 ± 1.7/1.5

Values are represented as means ± SD (upper/lower).

**Figure 4:**
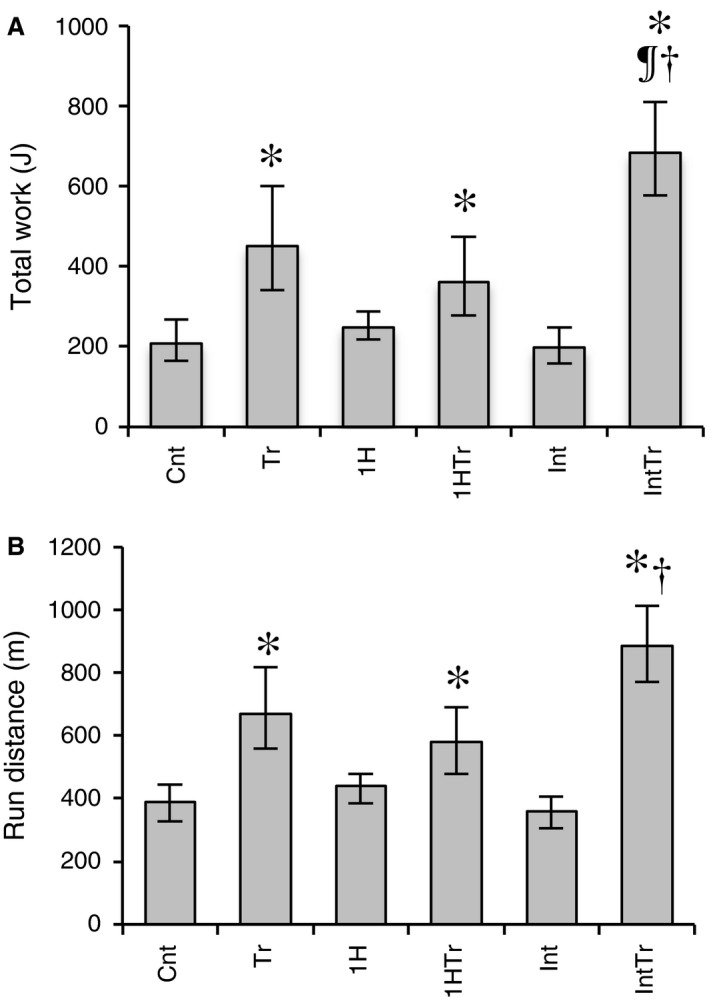
Total work capacity (A) and run distance (B) after 3 weeks of exercise training and/or hypoxic exposure. Values are represented as means ± SD. The number of mice was 12 per group. *, ^¶^, and ^†^, significantly different from the Cnt, Tr, and 1HTr groups, respectively, at *P* *<* 0.05 using a one‐way ANOVA followed by Tukey's post hoc test and 95% confidential interval did not contain the parameter value specified in the null hypothesis.

Maximal exercise capacity expressed in total work (joules) and run distance to exhaustion values were significantly greater in the Tr, 1HTr, and IntTr groups than in the Cnt group (*P* < 0.05, Fig. 4A and B). Moreover, total work values were significantly greater in the IntTr group than in the Tr and 1HTr groups (*P* < 0.05). Thus, short‐duration intermittent hypoxia had additive effects on exercise‐induced improvements in endurance exercise capacity.

#### Capillarization and muscle fiber profiles

**Figure 5:**
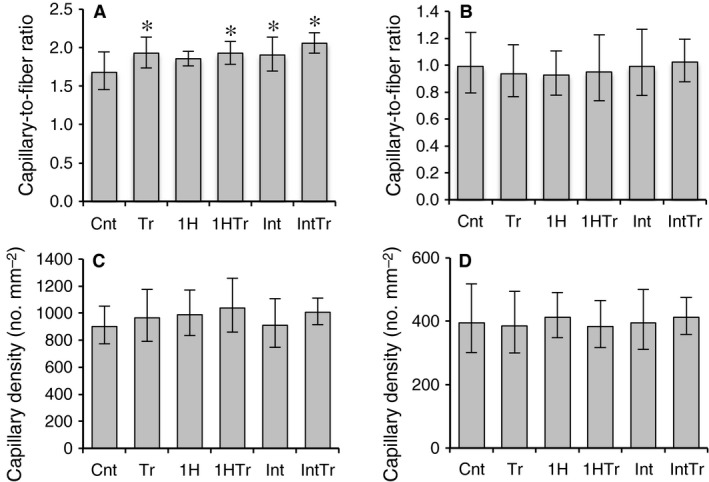
Capillary‐to‐fiber ratio (A, B) and capillary density (C, D) in the red (A, C) and white (B, D) regions of the gastrocnemius muscle after 3 weeks of exercise training and/or intermittent hypoxic exposure. Values are represented as means ± SD. The number of mice was 12 per group. *, significantly different from the Cnt group, at *P* *<* 0.05 using a one‐way ANOVA followed by Tukey's post hoc test and 95% confidential interval did not contain the parameter value specified in the null hypothesis.

The capillary‐to‐fiber (C:F) ratio in Gr was significantly greater in the Tr (by 14%), 1HTr (by 15%), Int (by 13%), and IntTr (by 22%) groups than in the Cnt group (Fig. 5A). The proportion of hybrid fibers increased in the IntTr group (by 27%) compared with the Cnt group, but the difference was not significant (Table 3). Thus, daily short‐duration intermittent hypoxic exposure did not facilitate exercise‐induced capillary growth in hindlimb muscles.

**Table 3:** Fiber cross‐sectional area (*μ*m^2^) and fiber type composition (%) values

	Cnt	Tr	1H	1HTr	Int	IntTr
Gr						
Slow						
Area	1951.7 ± 601.0/459.5	2012.6 ± 461.7/375.6	2025.4 ± 503.4/403.2	1842.2 ± 431.8/349.8	2128.1 ± 671.4/510.4	2114.9 ± 634.9/488.3
%	33.1 ± 3.4/3.1	28.9 ± 4.0/3.5	31.8 ± 2.3/2.2	32.6 ± 2.4/2.2	34.3 ± 2.7/2.5	35.3 ± 9.2/7.3
Fast						
Area	1905.0 ± 507.2/400.6	1977.8 ± 567.5/441.0	1690.6 ± 428.9/342.1	1721.7 ± 348.1/289.5	1986.1 ± 689.5/511.8	2082.5 ± 554.1/437.6
%	64.9 ± 3.3/3.1	66.3 ± 4.7/4.4	67.2 ± 2.7/2.6	66.7 ± 2.0/1.9	63.8 ± 2.8/2.7	62.6 ± 4.7/4.4
Slow + Fast						
Area	1348.6 ± 531.0/381.0	1291.6 ± 410.9/311.7	1256.6 ± 525.0/370.3	1170.8 ± 138.8/124.4	1353.1 ± 476.2/352.2	1569.5 ± 451.7/350.7
%	2.4 ± 0.20/0.18	2.9 ± 0.68/0.55	2.4 ± 0.34/0.30	2.1 ± 0.40/0.33	2.7 ± 0.47/0.40	3.1 ± 3.5/1.6
Gw						
Fast						
Area	2946.8 ± 840.9/654.2	2948.8 ± 906.0/693.1	2794.1 ± 661.5/534.9	2914.4 ± 787.5/620.0	2999.9 ± 387.7/343.3	3086.1 ± 589.0/494.6
%	100	100	100	100	100	100

Values are means ± SD (upper/lower).

#### Enzyme activity

**Figure 6:**
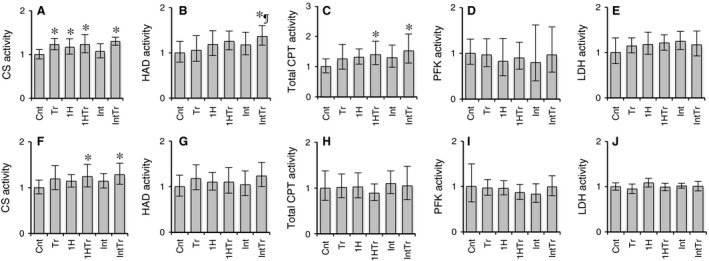
Relative enzyme activities (Cnt set to 1.0) in the red (A–E) and white (F–J) regions of the gastrocnemius muscle after 3 weeks of exercise training and/or intermittent hypoxic exposure. Values are represented as means ± SD. The number of mice was 12 per group. * and ^¶^, significantly different from the Cnt and Tr groups, respectively, at *P* *<* 0.05 using a one‐way ANOVA followed by Tukey's post hoc test and 95% confidential interval did not contain the parameter value specified in the null hypothesis. CS, citrate synthase; HAD, 3‐hydroxyacyl‐CoA‐dehydrogenase; CPT, carnitine palmitoyl transferase; PFK, phosphofructokinase; LDH, lactate dehydrogenase.

After exercise training, CS activity in Gr was significantly enhanced in the trained groups with and without hypoxic exposure (*P* < 0.05, Fig. 6A). CS activity in Gw was greater in the 1HTr and IntTr groups than in the Cnt group (*P* < 0.05, Fig. 6F). HAD activity in Gr were greater in the IntTr group than in the Cnt and Tr groups (*P* < 0.05, Fig. 6B). Total CPT activity in Gr was significantly increased in the 1HTr (by 40%) and IntTr (by 53%) groups (*P* < 0.05, Fig. 6C). Thus, exercise training with intermittent hypoxic exposure appears to be the more relevant methods to improve fatty acid metabolism in hindlimb muscles.

#### mRNA expression

**Figure 7:**
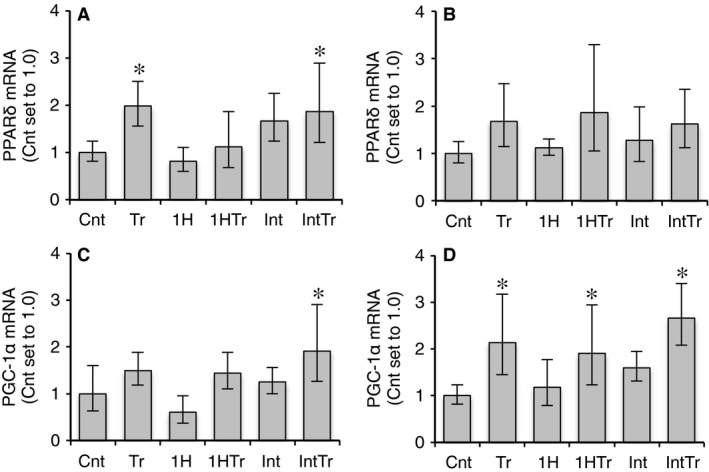
Expression of PPAR *δ* (A, B) and PGC‐1*α* (C, D) mRNA in the red (A, C) and white (B, D) regions of the gastrocnemius muscle after 3 weeks of exercise training and/or intermittent hypoxic exposure. Values are represented as means ± SD. The number of mice was 12 per group. *, significantly different from the Cnt group at *P* *<* 0.05 using a one‐way ANOVA followed by Tukey's post hoc test and 95% confidential interval did not contain the parameter value specified in the null hypothesis.

PPAR*δ* and PGC‐1*α* mRNA levels in Gr were both significantly greater in the IntTr group than in the Cnt group (*P* < 0.05, Fig. 7A and C). In the Tr groups, elevated PPAR*δ* and PGC‐1*α* mRNA levels were detected in Gr and Gw, respectively (*P* < 0.05, Fig. 7A and D).

## Discussion

The principal result of this study was that short‐duration intermittent hypoxic exposure mainly facilitated endurance exercise performance by promoting mitochondrial enzyme activities related to fatty acid metabolism in hindlimb muscles.

### Time‐course response to acute hypoxic exposure or exercise

Although HIF‐*α* subunits are constitutively expressed in cells, they are rapidly degraded under normoxic conditions by oxygen‐dependent prolyl hydroxylase domain (PHD)‐containing enzymes, which have been shown to require oxygen, iron, and 2‐oxyglutarate as cofactors (Ivan et al. 2001). PHDs are inactivated under hypoxic conditions, therefore HIF‐*α* subunits accumulate and translocate to the nucleus, in which they activate HIF‐responsive genes including EPO and VEGF‐A. In Hep 3B cells exposed to 1% O_2_ for 4 h, nuclear HIF‐1*α* protein levels were markedly increased immediately and 5 min post hypoxia, but were not detected 15 min post hypoxia (Wang et al. 1995). The expression of EPO mRNA in the kidney was markedly increased immediately after 1‐h hypoxia, intermittent hypoxia, or exercise, suggesting that transcriptional activation by the HIF protein was predominant immediately post hypoxia or exercise in this study. Enhanced VEGF‐A mRNA levels observed immediately after 1‐h hypoxia or exercise may have been due to the activation of HIF*α*, whereas the elevated levels observed 6 h post intermittent hypoxia may have been induced by HIF‐independent factors.

The expression of miR‐16, which was previously shown to suppress angiogenesis by inhibiting the expression of VEGF‐A (Sun et al. 2013), was markedly reduced after short‐duration intermittent hypoxia, but was not changed after 1‐h hypoxic exposure and exercise (Fig. 2C). Previous studies demonstrated that the expression of VEGF‐A was upregulated by PGC‐1*α* (Arany et al. 2008; Leick et al. 2009). VEGF‐A and PGC‐1*α* mRNA levels were both significantly greater 6 h post short‐duration intermittent hypoxia (Figs. 2 and 3). Thus, enhanced VEGF‐A mRNA expression after short‐duration intermittent hypoxia may be due to the upregulation of PGC‐1*α* and downregulation of miR‐16. The expression of miR‐23a, which has been shown to inhibit that of PGC‐1*α* (Russell et al. 2013), was significantly reduced after acute short‐duration intermittent hypoxia, suggesting that the upregulation of PGC‐1*α* after this exposure was due to the downregulated expression of miR‐23a. PGC‐1*α* has also been shown to stimulate energy production by activating the downstream genes involved in fatty acid and glucose metabolism in skeletal muscles (Benton et al. 2010). Therefore, acute short‐duration intermittent hypoxia may stimulate angiogenesis as well as mitochondrial biogenesis in hindlimb muscles.

### Chronic response of exercise training with intermittent hypoxic exposure

In this study, short‐duration intermittent hypoxia with training for 3 weeks facilitated endurance exercise capacity by enhancing mitochondrial enzyme activities related to fatty acid metabolism in hindlimb muscles. Although CS activity was significantly increased in Gr after training with or without intermittent hypoxia, training with intermittent hypoxia markedly enhanced HAD and total CPT activities (Fig. 6). Furthermore, HAD activity levels after training with intermittent hypoxia were significantly greater than those after training alone. mRNA levels for both PPAR*δ* and PGC‐1*α*, which improve fatty acid utilization in skeletal muscles (Vega et al. 2000; Dressel et al. 2003; Schuler et al. 2006), were significantly greater than sedentary control in Gr after training with short‐duration intermittent hypoxia, while only PPAR*δ* mRNA levels were enhanced after training (Fig. 7).

This study demonstrated, for the first time, that training with 1 h hypoxia and short‐duration intermittent hypoxia enhanced total CPT activity in hindlimb muscles. The CPT complex facilitates the entry of long‐chain fatty acids from the cytosol into the mitochondrial matrix, in which where beta‐oxidation occurs; therefore, it is considered to be a rate‐limiting enzyme of fatty acid utilization. Aerobic exercise training for 6 weeks markedly increased CPT‐1 mRNA and protein levels in mice skeletal muscle (Niu et al. 2010), and these changes were shown to be regulated in part by myocyte‐specific enhancer factor (Yuan et al. 2014). After 5 weeks of hypobaric hypoxia (simulated 4000 m), muscle CPT‐1 activity was shown to be markedly decreased in rat quadriceps muscles, whereas these activity levels reached those of normoxic sedentary controls after exercise training under hypoxia, which indicated that exercise training restored muscle CPT activity in the hypoxic environment (Galbès et al. 2008). In this study, 3 weeks of endurance training alone slightly increased total CPT activity (by 1.3 fold, 95% CI: 1.03–1.6 fold, *P* = 0.29, Fig. 6C), whereas training with 1 h hypoxia (by 1.4 fold, 95% CI: 1.2–1.7 fold, *P* = 0.037) and short‐duration intermittent hypoxia (by 1.5 fold, 95% CI: 1.3–1.9 fold, *P* = 0.004) markedly increased this activity. Furthermore, training with short‐duration intermittent hypoxia enhanced both total CPT and HAD activity levels. Thus, exercise training with short‐duration intermittent hypoxia may facilitate mitochondrial fatty acid metabolism during exercise, thereby improving maximal endurance capacity.

Acute short‐term intermittent hypoxia up‐regulated PGC‐1*α* mRNA levels (Fig. 3), which has been shown to induce angiogenesis (Arany et al. 2008; Leick et al. 2009). The C:F ratio values were markedly increased after chronic short‐duration intermittent hypoxia than in sedentary control group. However, the C:F ratio values were not significantly different between the Tr and IntTr (Fig. 5). Thus, chronic exposure to intermittent hypoxia may promote capillary growth but did not facilitate exercise‐induced capillary growth in hindlimb muscles.

In conclusion, this study demonstrated that exercise training with short‐duration intermittent hypoxia for 3 weeks clearly had additive effects on training‐induced increases in endurance performance. HAD and total CPT activities were markedly increased after training with short‐duration intermittent hypoxia. The present findings show that exercise training under normoxic conditions with short‐duration intermittent hypoxia represents a beneficial strategy for increasing endurance performance by enhancing mitochondrial enzyme activities concerning fatty acid metabolism in skeletal muscles.

## Conflict of Interests

None declared.
